# Trypanocidal and Anti-Inflammatory Effects of Three *ent*-Kaurane Diterpenoids from *Gymnocoronis spilanthoides* var. *subcordata* (Asteraceae)

**DOI:** 10.3390/pharmaceutics16030415

**Published:** 2024-03-18

**Authors:** Mariana G. Selener, Jimena Borgo, Maria Belen Sarratea, Maria Alicia Delfino, Laura C. Laurella, Natacha Cerny, Jessica Gomez, Mauro Coll, Emilio L. Malchiodi, Augusto E. Bivona, Patricia Barrera, Flavia C. Redko, César A. N. Catalán, Andrés Sánchez Alberti, Valeria P. Sülsen

**Affiliations:** 1 Cátedra de Farmacognosia, Facultad de Farmacia y Bioquímica, Universidad de Buenos Aires, Junín 956, Piso 2, Buenos Aires C1113AAD, Argentina; m.selener@docente.ffyb.uba.ar (M.G.S.); jborgo@docente.ffyb.uba.ar (J.B.); c.laurella@docente.ffyb.uba.ar (L.C.L.); fredko@ffyb.uba.ar (F.C.R.); 2 Instituto de Química y Metabolismo del Fármaco (IQUIMEFA), CONICET-Universidad de Buenos Aires, Junín 956, Piso 2, Buenos Aires C1113AAD, Argentina; 3 Instituto de Estudios de la Inmunidad Humoral (IDEHU), CONICET-Universidad de Buenos Aires, Junín 956, Piso 4, Buenos Aires C1113AAD, Argentina; mbsarratea@ffyb.uba.ar (M.B.S.); emalchio@ffyb.uba.ar (E.L.M.); aebivona@ffyb.uba.ar (A.E.B.); 4 Cátedra de Inmunología, Facultad de Farmacia y Bioquímica, Universidad de Buenos Aires, Junín 956, Piso 4, Buenos Aires C1113AAD, Argentina; madelfino@docente.ffyb.uba.ar; 5 Departamento de Microbiología, Parasitología e Inmunología-IMPAM (UBA-CONICET), Facultad de Medicina, Universidad de Buenos Aires, Paraguay 2155, Buenos Aires C1121ABG, Argentina; ncerny@docente.ffyb.uba.ar; 6 Facultad de Ciencias Médicas, Instituto de Histología y Embriología “Dr. Mario H. Burgos” (IHEM), Universidad Nacional de Cuyo-CONICET, CC 56, Mendoza 5500, Argentina; jgomez@mendoza-conicet.gob.ar (J.G.); mauro.coll16@gmail.com (M.C.); pbarrera@mendoza-conicet.gob.ar (P.B.); 7 Instituto de Química Orgánica, Facultad de Bioquímica, Química y Farmacia, Universidad Nacional de Tucumán, Ayacucho 471, San Miguel de Tucumán T4000INI, Argentina; cancatalan@gmail.com

**Keywords:** *Gymnocoronis*, diterpene, natural compounds, *ent*-kaurane, *Trypanosoma cruzi*, anti-inflammatory activity, antiparasitic

## Abstract

Chagas disease, caused by the protozoan *Trypanosoma cruzi*, affects 6–7 million people worldwide. The dichloromethane extract obtained from the aerial parts of *Gymnocoronis spilanthoides* var *subcordata* showed trypanocidal activity in vitro. The fractionation of the dewaxed organic extract via column chromatography led to the isolation of three diterpenoids: *ent*-9α,11α-dihydroxy-15-oxo-kaur-16-en-19-oic acid or adenostemmoic acid B, (16*R*)-*ent*-11α-hydroxy-15-oxokauran-19-oic acid and *ent*-11α-hydroxy-15-oxo-kaur-16-en-19-oic acid. These compounds showed IC_50_ values of 10.6, 15.9 and 4.8 µM against *T. cruzi* epimastigotes, respectively. When tested against amastigotes, the diterpenoids afforded IC_50_ values of 6.1, 19.5 and 60.6 µM, respectively. The cytotoxicity of the compounds was tested on mammalian cells using an MTT assay, resulting in CC_50_s of 321.8, 23.3 and 14.8 µM, respectively. The effect of adenostemmoic acid B on *T. cruzi* was examined at the ultrastructural level using transmission microscopy. Treatment with 20 μM for 48 h stimulated the formation of abnormal cytosolic membranous structures in the parasite. This compound also showed an anti-inflammatory effect in murine macrophages stimulated with LPS and other TLR agonists. Treatment of macrophages with adenostemmoic acid B was able to reduce TNF secretion and nitric oxide production, while increasing IL-10 production. The combination of adenostemmoic acid B with benznidazole resulted in greater inhibition of NF-kB and a decrease in nitrite concentration. The administration of adenostemmoic acid B to mice infected with trypomastigotes of *T. cruzi* at the dose of 1 mg/kg/day for five days produced a significant decrease in parasitemia levels and weight loss. Treatment with the association with benznidazole increased the survival time of the animals. In view of these results, adenostemmoic acid B could be considered a promising candidate for further studies in the search for new treatments for Chagas disease.

## 1. Introduction

Chagas’ disease or American trypanosomiasis is an infectious disease caused by the protozoan *Trypanosoma cruzi*. It is categorized among the 20 neglected tropical diseases by the World Health Organization, primarily affecting poor communities in tropical regions [1]. The disease is prevalent in 21 countries in Latin America and impacts around 6–7 million individuals worldwide, leading to severe health, social, and economic repercussions [2]. The most relevant transmission routes for this parasite include the following: I Vector-borne transmission, caused by the contamination of skin wounds or mucous membranes which occurs when hematophagous insects from the *Triatominae* subfamily feed from the mammalian hosts; II Vertical transmission, caused by the passage of the parasite across the placenta; III Transmission through organ or blood donations; IV Transmission through the ingestion of contaminated food or water [3].

The life cycle of *T. cruzi* involves an invertebrate host (the triatomine vector insect) and a vertebrate mammalian host for its completion, including humans among the latter. *T. cruzi* has four stages with differential functional, morphological and structural characteristics. The epimastigote stage is the replicative, non-infective form found in the midgut of the vector insect. Epimastigotes differentiate into metacyclic trypomastigotes in the distal gut of the vector, representing a non-replicative, infective form that is deposited in the feces of the triatomine to infect humans through lesions in the skin or intact mucous membranes, mainly of the conjunctiva. Once inside the cells, both metacyclic and bloodstream trypomastigotes differentiate into the replicative intracellular form called the amastigote. Lastly, amastigotes differentiate into bloodstream trypomastigotes, which lyse infected cells and access the bloodstream to infect new cells or a new vector to restart the parasite’s biological cycle. This stage is characterized by being non-replicative and infective for the vector insect and mammalian host [4].

Current medications for the treatment of this parasitic infection include benznidazole and nifurtimox, which are effective during the acute phase of the disease. The benefits are unclear regarding the treatment with these drugs in the chronic phase. Although studies published in the past years indicate that treatment manages to reduce the parasitic load, as reflected in PCR negativization, the lack of improvement in the cardiomyopathy frequently associated with the chronic phase is discouraging [5,6]. Moreover, benznidazole and nifurtimox present a high rate of undesirable side effects. Consequently, there is an urgent need for the development of new anti-*T. cruzi* treatments [7].

The innate immune system plays a crucial role in the initial defense against *T. cruzi*. Various pathogen recognition receptors (PRRs) can be activated during infection with *T. cruzi*. In particular, Toll-like receptors (TLRs) have been identified as contributors to orchestrating this response, including TLR2, TLR3, TLR4 and TLR9 which are involved during the early stage of the infection [8]. The signaling pathways of TLRs via MyD88 culminate in activation of the nuclear factor-kappaB (NF-kB) transcription factor, which regulates the expression of a spectrum of inflammatory cytokine genes [9]. In relation to the adaptive immune response, disease control during the acute phase is dependent on antibodies, CD8+ lymphocytes (cytotoxic), and CD4+ lymphocytes (helper), specifically the Th1 type that produces high levels of IFN-γ. The immune system appears to have a modulatory effect on cardiac function during the development of chronic Chagasic cardiomyopathy (CCC). Patients with CCC have increased levels of circulating or heart TNF compared to individuals in the undetermined phase of the disease. Additionally, an association between heart cells producing TNF and IFN-γ and poor cardiac function has been demonstrated in these patients [10]. On the other hand, the anti-inflammatory cytokine IL-10 is involved in delaying the onset of CCC in infected individuals. A comprehensive analysis of the cytokine profile has revealed that a reduction in IL-10 levels alters the immune response from the anti-inflammatory profile seen in asymptomatic patients compared to that observed in cardiac patients [11]. In this sense, modulating the inflammatory response in individuals with Chagas disease could have therapeutic potential [12].

Natural products represent a wide source of molecules with high structural diversity which have been associated with several biological activities and therefore constitute interesting candidates in the drug discovery process [13]. In this sense, the potential of natural products as antiparasitic agents has been demonstrated by the development of ivermectin, artemisinin, quinine and their derivatives, used for the treatment of neglected diseases mainly in developing countries. A vast number of phytochemicals, including terpenoids, alkaloids and phenolic compounds, have been pointed out as promising candidates for the development of drugs against Chagas disease [14,15]. 

*Gymnocoronis spilanthoides* var. *subcordata* (Asteraceae) is a freshwater aquatic plant native to South America. This species is popularly known as Senegal tea plant. In South America, it is called “Jazmín del bañado”. Given its ornamental uses, *G. spilanthoides* var. *subcordata* was initially introduced as an aquarium and aquatic ornamental plant in several countries [16,17]. However, it rapidly became an invasive alien in all the regions where it was introduced, including Asia, Australia, New Zealand, Europe, China, Japan and Taiwan. In 2012, *G. spilanthoides* was included on the “EPPO Observation List of invasive alien plants” by the European and Mediterranean Plant Protection Organization [17,18]. Regarding the chemical composition of *G. spilanthoides*, several pyrrolizidine alkaloid esters such as intermedine, lycopsamine gymnocoronine and others have been characterized via HPLC–ESI(+)/MS and MS/MS analysis of the methanolic extract of this species, comparing the spectra and fragmentation pattern of itsconstituents with data found in the literature [17]. Nevertheless, to our knowledge, the major phytochemical components of this species have not been studied. We have previously reported that the dichloromethane extract obtained from the aerial parts of *G. spilanthoides* showed in vitro trypanocidal activity on *Trypanosoma cruzi* [19]. Considering the easy propagation and invasive behavior of *G. spilanthoides*, along with the previously reported trypanocidal activity and its unknown major constituents, this Asteraceae represents an interesting candidate as a potential source of trypanocidal compounds.

In this paper, we aim to isolate the bioactive phytochemicals from *G. spilanthoides* var. *subcordata* and evaluate the anti-inflammatory effects of the most active and selective compound in the search for novel molecules with potential for the development of new treatments for Chagas’ disease.

## 2. Materials and Methods

### 2.1. Plant Material

The aerial parts of *Gymnocoronis spilanthoides* (D. Don ex Hook. & Arn.) DC. var. *subcordata* (BAF787) were collected in Entre Ríos Province, Argentina, in February 2012. A voucher specimen is deposited at Museo de Farmacobotánica, Facultad de Farmacia y Bioquímica, Universidad de Buenos Aires.

### 2.2. Isolation and Purification of Phytochemicals from G. spilanthoides var. subcordata

Once air-dried at room temperature protected from sunlight and humidity, the aerial parts of *G. spilanthoides* var. *subcordata* (560 g) were extracted twice via maceration with dichloromethane (4 L) for 24 h. The resulting extracts were reunited and taken to dryness in a rotary evaporator in order to obtain the initial dichloromethane extract from which the yield was calculated [extract yield (%) = 0.6 (dry dichloromethane extract (g) × 100)/(dry aerial parts of *G. spilanthoides*)]. The extract was subjected to a dewaxing process according to the protocol outlined by Elso et al., 2020 [20] and afterward fractionated using column chromatography on Silicagel (55 × 3 cm, 100 g, 230–400 mesh) with a dichloromethane: ethyl acetate gradient (DCM:EtOAc) (100:0–0:100). A total of 70 fractions of 15 mL each were collected and three precipitates were obtained in the fractionation process. The precipitates were washed repeatedly with ethyl acetate. Fractions 30–32, eluted with DCM: EtOAc (3:1), afforded a precipitate named compound 1. From fractions 33–37 [eluted with DCM: EtOAc (2:1)], and from fractions 38–41 [eluted with DCM: EtOAc (2:1)], compound 2 and 3 were isolated. 

### 2.3. Purity of the Isolated Compounds

The chromatographic behavior of the compounds was assessed using thin layer chromatography on three chromatographic systems with Silicagel 60 F_254_ as stationary phase and n-hexane: EtOAc (5:5) (system I), DCM: acetone (7:3) (system II) and toluene: acetone (6:4) (system III) as mobile phases and anisaldehyde sulfuric acid as a developing reagent.

A preparative HPLC coupled to a photodiode array detector (PDA-HPLC) (Waters 600/PDA Waters 2996) was used for the purification of compound 2. The instrument was equipped with a reversed-phase column Phenomenex Luna C18 (250 mm × 10 mm and 10 μm dp) and a Rheodyne valve (100 µL). Samples were eluted with a gradient of water (A) and acetonitrile (B) from 37% B to 50% B in 20 min. The flow rate was set at 3.2 mL/min. 

The final purity of compounds 1–3 was determined by a PDA-HPLC using an Agilent 1260 instrument, equipped with a reversed-phase column Phenomenex Luna C18 (250 mm × 4.6 mm and 5 μm dp). Solutions of the compounds dissolved in methanol at 1 mg/mL were prepared for the analysis. A 50 µL injection volume was used. The elution was carried out with a gradient of water (A) and acetonitrile (B) from 50% B to 98% B in 15 min. The flow rate was set at 1 mL/min and the UV detector at 215 nm.

### 2.4. Structure Elucidation of the Isolated Compounds

The identification of compounds 1–3 was determined via proton nuclear magnetic resonance (^1^H-NMR) and carbon nuclear magnetic resonance (^13^C-NMR), heteronuclear single quantum correlation (HSQC), heteronuclear multiple bond correlation (HMBC), correlated spectroscopy (COSY) (Bruker Avance III 600 MHz, Bruker AG, Rheinstetten, Germany) (600 MHz in CDCl_3_), electron impact-mass spectrometry (EI-MS) (Thermo Scientific EM/DSQ II, Thermo Fisher Scientific Inc., Waltham, MA, USA; Shimadzu QP5000, Shimadzu Corporation, Nakagyo-ku, Kyoto, Japan) and infrared spectroscopy (IR) (Shimadzu Corporation, Nakagyo-ku, Kyoto, Japan) by comparing the spectra obtained with those found in the literature. 

### 2.5. Trypanosoma cruzi In Vitro Testing

#### 2.5.1. Parasites

Cultures of *Trypanosoma cruzi* epimastigotes (RA strain) were maintained through weekly passages in LIT medium supplemented with 10% fetal bovine serum at 28 °C. *Trypanosoma cruzi* bloodstream trypomastigotes (RA strain) were obtained from infected CF1 mice by cardiac puncture 15 days after infection at the peak of parasitemia while transfected trypomastigotes expressing β-galactosidase (Clone C4, e Tulahuen strain) were obtained from infected Vero cells [21].

#### 2.5.2. In Vitro Activity Assay against Epimastigotes

The [^3^H]-thymidine uptake assay was implemented to determine the growth inhibition of *T. cruzi* epimastigotes in the presence of the phytochemicals isolated from *G. spilanthoides* var. *subcordata* [22]. Cell density was adjusted to 1.5 × 10^6^ parasites/mL with BHT medium. compounds 1–3 were dissolved in DMSO to be tested at concentrations of 1–100 µg/mL. Benznidazole was used as a positive control, while DMSO was included as a negative control for the vehicle. After incubation for 72 h, radioactivity was measured as counts per minute (cpm). The percentage of inhibition was calculated as: 100 − {[(cpm of treated parasites)/(cpm of untreated parasites)] × 100}.

#### 2.5.3. In Vitro Activity Assay against Amastigotes

The activity of compounds 1–3 on the intracellular forms of *T. cruzi* was evaluated [23]. Vero cells (5 × 10^3^/well) were infected with culture-derived trypomastigotes of *T. cruzi* from the Tulahuen strain expressing β-galactosidase (MOI: 10). After an O.N. incubation at 37 °C, and 5% CO_2_, non-infecting parasites were washed out and infected cells were incubated with increasing concentrations of the compounds (0.001–50 µg/mL). Cells treated with DMSO or benznidazole and non-infected cells were included as controls. After 5 days, cells were lysed with 1% Nonidet P-40, and chlorophenol red-β-D-galactopyranoside (CPRG) (100 μM) was added as a substrate for the β-galactosidase enzyme. The plate was then incubated for 4 h at 37 °C and β-galactosidase activity was determined by measuring the absorbance at 570 nm. The percentage of inhibition for each concentration of compounds was calculated as: 100 − {[(absorbance of treated infected cells)/(absorbance of untreated infected cells)] × 100}.

#### 2.5.4. In Vitro Activity Assay against Trypomastigotes

The survival of bloodstream trypomastigotes from *T. cruzi* after treatment with the isolated compounds was evaluated as previously described [23]. Mouse blood containing trypomastigotes was diluted in RPMI medium to a cell density of 1.5 × 10^6^ parasites/mL. Parasites were incubated with different concentrations of compounds 1–3 (1–100 µg/mL) for 24 h at 37 °C. DMSO and benznidazole were used as negative and positive controls, respectively. Live trypomastigotes were counted in a Neubauer chamber. The percentage of live trypomastigotes was calculated as: {[(live parasites in treated wells)/(live parasites in untreated wells)] × 100}.

### 2.6. Cytotoxicity Assay

The cytotoxicity of the isolated compounds was evaluated using splenocytes obtained from BALB/c mice [24]. Primary cell cultures (1.5 × 10^5^) were incubated with different concentrations of the isolated phytochemicals (5–200 μg/mL) in RPMI medium supplemented with 10% fetal bovine serum. After 48 h of incubation at 37 °C (5% CO_2_), the cells were harvested, washed once with PBS and stained with 2.5 μg/mL propidium iodide (PI) for 5 min at room temperature. Subsequently, cell death was assessed via flow cytometry using a BD FACSaria II cytometer. Cells incubated with DMSO were used as 100% viability control, and the death percentage was calculated according to the following formula: {1 − [(% PI^−^ cells treated)/(% PI^−^ cells of the viability control)] × 100}.

### 2.7. Transmission Electron Microscopy

All procedures were carried out according to Brengio et al. [24]. Epimastigotes from *T. cruzi* were treated with 20 µM of compound 1 for either 48 h or 72 h. Then, the parasites were centrifuged at 1000× *g* for 10 min and fixed with 2.5% glutaraldehyde. Subsequently, they were washed three times with PBS and postfixed overnight with 2% OsO_4_. After washing twice with PBS, cells were stained with 1% uranyl acetate. The samples were dehydrated sequentially in ethanol and acetone and embedded in Epon 812. Ultrathin slices of 60 nm were obtained using an automatic Leica-ultracut R ultramicrotome and observed with a Microscope Zeiss EM900 (Zeiss, Oberkochen, Germany) [25].

### 2.8. Anti-Inflammatory Properties of Compound *1*

#### 2.8.1. Effect of Compound 1 on the Type-I Interferon Pathway

The effect of compound 1 on a type-I Interferon (IFN) reporter cell line stimulated with LPS was studied. RAW-Lucia cells express the Lucia luciferase enzyme under the control of the ISG54 promoter that responds to an IFN-stimulated response element, which is activated by several Interferon Regulatory Factors (IRFs) [26].

RAW-Lucia ISG cells I (InvivoGen, San Diego, CA, USA) (100 μL) were cultured (100,000 cells/well) with DMEM at 37 °C in a 5% CO_2_ atmosphere to be stimulated with LPS (0.5 µg/mL) or a combination of LPS and compound 1 (1 and 10 µg/mL). Unstimulated cells were included as controls. After a 24 h incubation, the supernatants of each well were collected in order to quantify the luciferase activity by measuring the luminescence generated after the addition of the *QUANTI-Luc* reagent (InvivoGen) according to the manufacturer’s instructions with a PerkinElmer Victor3 luminometer.

The viability of the stimulated cells was tested using the XTT assay (Roche, Mannheim, Germany). After incubation for 24 h with LPS and compound 1, fresh medium was added with 0.2 mg/mL XTT and 0.5 µg/mL PMS. After 2 h at 37 °C, absorbance was measured at 450 nm with correction at 620 nm in a microplate reader.

#### 2.8.2. Effect of Compound 1 on the Secretion of NF-kB

The effect of compound 1 was evaluated on a cell line that secretes alkaline phosphatase as a surrogate marker for the secretion of NF-kB. RAW-Blue cells (InvivoGen) (100 μL) were seeded in a 96-well plate (100,000 cells/well) with DMEM at 37 °C in a 5% CO_2_ atmosphere. Combinations of compound 1 (1 and 10 μg/mL) and LPS (0.5 μg/mL), compound 1 (2.5 µg/mL) with a TLR (Toll-Like Receptor) agonist [Cytosine-Guanine dinucleotides (CpG) (100 μg/mL) or polyinosinic-polycytidylic acid (poly(I:C) (100 μg/mL) or LPS (0.5 μg/mL)], or compound 1 (2.5 µg/mL) with LPS (0.5 μg/mL) and Benznidazole (100 µM) were added, followed by a 24 h incubation period. Untreated cells were included as controls. The supernatants were collected and absorbance at 620–655 nm was measured after the addition of the Quanti-Blue reagent (InvivoGen) in a microplate reader. Levels of NF-kB were calculated following the manufacturer’s instructions.

The production of the cytokines IL-10 and TNF was also determined in cell supernatant from these samples with an ELISA assay (R&D System, Minneapolis, MN, USA; BD Pharmingen, Franklin Lakes, NJ, USA). 

#### 2.8.3. Nitric Oxide Production in the Presence of Compound 1

The Griess reaction assay was performed to determine the effect of compound 1 on Nitric Oxide production [27]. The same supernatant samples obtained in Section 2.8.2 (50 µL) were combined with 100 µL of Griess reagent. After incubation at room temperature for 30 min, absorbance was measured at 540 nm. A calibration curve was constructed with 1–250 µM of sodium nitrate to extrapolate the nitrite concentration in the tested samples. 

### 2.9. Trypanosoma cruzi In Vivo Testing

#### 2.9.1. Mice

Inbred BALB/c mice (female, 6–8 weeks old) were kept at the animal care facility of Instituto de Microbiología y Parasitología Médica, IMPaM, Universidad de Buenos Aires-CONICET. Animal experiments were approved by the Review Board of Ethics of Universidad de Buenos Aires, Facultad de Medicina, Argentina (no. 2943/2013). All procedures were performed following the guidelines established by the National Research Council [28]. 

#### 2.9.2. In Vivo Trypanocidal Activity

The mice were infected with 1000 bloodstream trypomastigotes of the RA strain via intraperitoneal inoculation. Parasitemia was measured three times a week. Blood samples (5 µL) were diluted 1:5 in lysis buffer (0.75% NH_4_Cl, 0.2% Tris, pH 7.2), and parasites were counted in a Neubauer chamber.

Mice were divided into groups of five animals each, and the drugs were administered via the intraperitoneal route (1 mg/kg of body weight/day) for five consecutive days after infection (dpi) (4–8 dpi). compound 1 and benznidazole were diluted with DMSO, and the concentration was adjusted with 0.1 M phosphate-buffered saline (pH 7.2). The vehicle was employed as a negative control. The efficacy of treatment was evaluated by recording the weight of the animals, their survival and the measured parasitemia throughout the experiment.

### 2.10. Statistical Analysis

The results shown are presented as mean ± SEM. GraphPad Software, Inc., (San Diego, CA, USA) was used for determination of the 50% inhibitory concentration (IC_50_) and the 50% cytotoxic concentration (CC_50_). Statistical significance was determined with the software employing one-way or two-way ANOVA and the indicated post-test in each figure. Comparisons were referred to the control group unless otherwise indicated. *p* values < 0.05 were considered significant.

## 3. Results

### 3.1. Isolated Phytochemicals from G. spilanthoides var. subcordata

Structural elucidation of compounds 1–3 was performed using spectroscopic methods, comparing the experimental data with spectra found in the literature [29,30]. 

compound 1 showed a molecular formula C_20_H_28_O_5_. The IR spectrum displayed diagnostic absorptions at 3300–2500 cm^−1^ (OH), 1720 cm^−1^ (cyclopentanone), 1690 cm^−1^ (carboxyl carbonyl) and 1640 cm^−1^ (conjugated double bond). The ^1^H-NMR spectrum in DMSO-d_6_ at 600 MHz (Table 1) showed two *exo*-methylene protons conjugated to a carbonyl group at δ 5.52 (s) and 5.15 (s) assigned to H-17a and H-17b, respectively, two tertiary methyls at δ 1.12 (Me-18) and 1.01 (Me-20), a carbinol proton at δ 3.77 (dd, J ~ 3.8 and 2 Hz; H-11) and distinctive signals at δ 2.92 (m; H-13) and 2.53 (d, 12 Hz; H-14a), while the ^13^C-NMR spectrum displayed twenty carbon signals (Table 1) that unveiled the structure as that corresponding to adenostemmoic acid B (*ent*-9α,11α-dihydroxy-15-oxo-kaur-16-en-19-oic acid) [21]. All assignments were performed using 2D experiments (H-H COSY, HSQC, HMBC) while the relative configuration was established through coupling constants values and fundamentally through NOESY correlations as shown in Table 1. Thus, in the NOESY spectrum, H-11 (equatorial) correlated with H-1b (equatorial) and H-14a (axial) correlated with H-20 (Me-20 axial). Comparing the assignments of Table 1 with those reported by Shimizu et al. [29] for adenostemmoic acid B and taking into account the different solvents used (DMSO-d6 vs. pyridine-d5), it is clear that the signals for C-1, C-7 and C-14 were incorrectly assigned and must be revised as shown here in Table 1. The spectra of compound 1 were included as Supplementary Materials.

compound 2 was identified as (16*R*)-*ent*-11α-hydroxy-15-oxokauran-19-oic acid. The stereochemistry of the chiral center was deduced using the magnitude of J_13,16_. The ^1^H-NMR data of 2 are in agreement with those reported in the literature [30]; its previously unreported ^13^C-NMR data are presented in Table 2. The ^13^C-NMR spectrum of 11α-acetate methyl ester derivative of 2 was reported by Herz and Sharma, 1976 [30].

The HRMS of compound 3 indicated a molecular formula C_20_H_30_O_4_ having an oxygen atom less than 1. The ^1^H- and ^13^C-NMR spectra of 3 established its structure as *ent*-11α-hydroxy-15-oxo-kaur-16-en-19-oic acid. NMR data of 3 are summarized in Table 2. The ^13^C-NMR assignments of 3 are in excellent agreement with those reported by Herz and Sharma in 1976 [30], except for the signals corresponding to C-7 and C-14 that should be exchanged as shown in Table 2. The spectra of compound 2 and 3 were included as Supplementary Materials.

**Figure 1:**
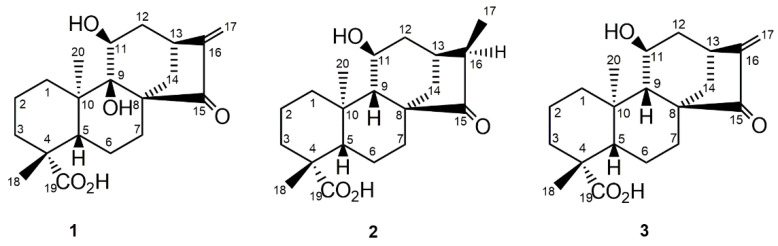
Chemical structures of phytochemicals isolated from *G. spilanthoides* var. *subcordata*. *Ent*-9α,11α-dihydroxy-15-oxo-kaur-16-en-19-oic acid or adenostemmoic acid B (1), (16*R*)-*ent*-11α-hydroxy-15-oxokauran-19-oic acid (2) and *ent*-11α-hydroxy-15-oxo-kaur-16-en-19-oic acid (3).

The structures of the isolated phytochemicals *ent*-9α,11α-dihydroxy-15-oxo-kaur-16-en-19-oic acid or adenostemmoic acid B (compound 1), (16*R*)-*ent*-11α-hydroxy-15-oxokauran-19-oic acid (compound 2) and *ent*-11α-hydroxy-15-oxo-kaur-16-en-19-oic acid (compound 3) are shown in Figure 1.

The isolated compounds were assessed using PDA-HPLC, resulting in purity levels of 97.1, 99.0 and 97.5% for compounds 1–3, respectively. The yield of these phytochemicals, calculated as yield (%) = {[pure compound 1–3 (mg) × 100]/crude dichloromethane extract of *G. spilanthoides* (mg)}, was as follows: 2.1 (31 mg), 3.7 (55 mg) and 2.3% (34 mg), respectively.

### 3.2. In Vitro Assays against Trypanosoma cruzi

**Figure 2:**
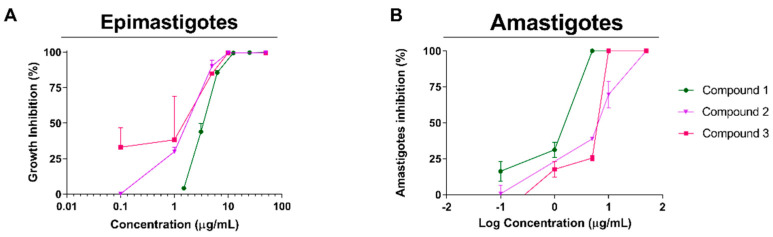
Effect of compounds 1–3 on the epimastigote and amastigote stages of *T. cruzi*. Every concentration was tested in triplicate to evaluate the inhibition of *T. cruzi* epimastigotes (**A**) and amastigotes (**B**). Results are expressed as the mean ± SEM.

The effect of the isolated diterpenes was evaluated in vitro against epimastigotes, trypomastigotes and amastigotes of *T. cruzi* (Figure 2, Table 3). 

compounds 1–3 were active against epimastigotes, reaching IC_50_ values of 3.7 ± 0.5, 5.2 ± 0.3 and 1.6 ± 0.6 µg/mL (10.6, 15.9 and 4.8 µM), respectively. When tested against amastigotes, compounds 1–3 showed activity against the intracellular form of the parasite with IC_50_ values of 2.1 ± 0.3, 6.5 ± 0.2 and 20.1 ± 8.2 µg/mL (6.1, 19.5 and 60.6 µM), respectively (Table 1). Results of the effect of the reference drug benznidazole on both forms of *T. cruzi* are also shown in Table 3. The three compounds were shown to be inactive against bloodstream trypomastigotes of *T. cruzi*.

### 3.3. Cytotoxicity and Selectivity against Trypanosoma cruzi

**Figure 3:**
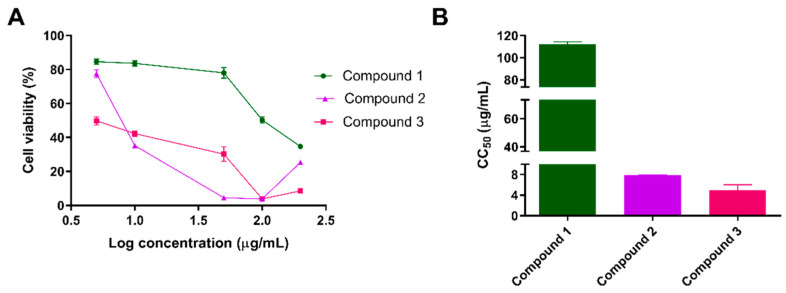
Cytotoxicity evaluation of the isolated compounds on murine splenocytes. Cells were incubated with increasing concentrations of the compounds, tested in duplicate. (**A**) Percentage of cell viability in the presence of compounds 1–3. (**B**) Calculated CC_50_ for each compound. Bars represent the means ± SEM.

The cytotoxic effect of compounds 1–3 was assessed in vitro on splenocytes. The CC_50_ values were 112.0 ± 3.4, 7.8 ± 0.2 and 4.9 ± 1.6 µg/mL (321.8, 23.3 and 14.8 µM) (Figure 3, Table 4). compound 1 was shown to be highly selective against the intracellular form of *T. cruzi*. Its selectivity index (SI), calculated as the ratio between CC_50_ and IC_50_, was 52.7. The SIs calculated for each compound can be seen in Table 4.

### 3.4. Transmission Electron Microscopy

**Figure 4:**
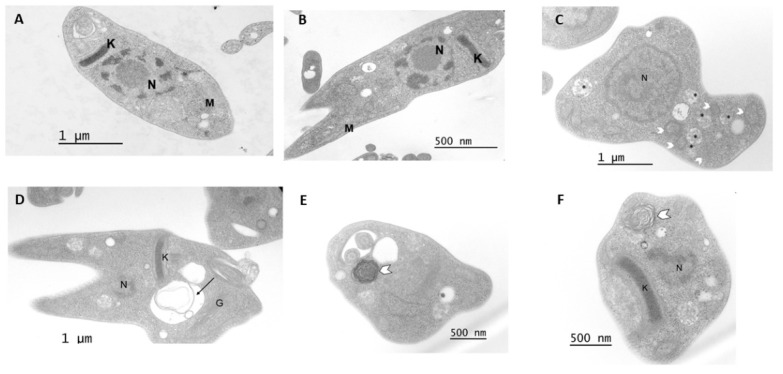
(**A**,**B**) Untreated parasites displayed typical morphology after a 48 h incubation (**C**–**F**) Epimastigotes treated with compound 1 (20 µM, 48 h). Parasites showed the formation of mitochondrial branches (arrowheads) and abnormal cytosolic membranous structures (arrow). N: nucleus, K: kinetoplast, M: mitochondrion, G: Golgi; glycosomes (asterisk).

**Figure 5:**
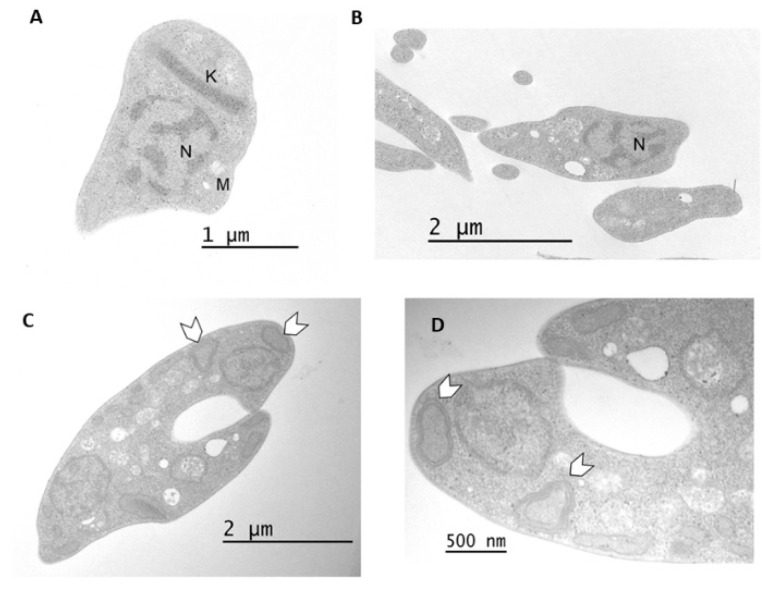
(**A**,**B**) Non-treated parasites showed typical morphology after a 72 h incubation period. (**C**,**D**) Epimastigotes treated with compound 1 (20 µM, 72 h). Abnormal cytosolic membranous structures (white arrowheads) appeared. N: nucleus, K: kinetoplast, M: mitochondrion.

The effect of compound 1 at the ultrastructural level of *T. cruzi* was analyzed in epimastigotes using transmission electron microscopy. Untreated parasites displayed typical morphology: a single nucleus, a kinetoplast near the flagellum and a single branched mitochondrion (Figure 4A,B and Figure 5A,B). After 48 h of treatment with 20 µM of compound 1, the formation of abnormal cytosolic membranous structures was observed (Figure 4C–F). Similar phenotypes were observed at 72 h after treatment (Figure 5).

### 3.5. Anti-Inflammatory Activity of Compound *1*

Considering that compound 1 showed the best performance in terms of selectivity as an anti-*T cruzi* drug, we decided to further analyze the anti-inflammatory activity of this molecule in vitro. To that end, the effect of the compound was assessed on Raw-Lucia cells which constitute a reporter cell line of IFN-I through the expression of the enzyme Lucia luciferase. Raw-Lucia cells were cultured with different concentrations of compound 1 after stimulation with LPS, a TLR4 agonist, which activates the IFN-I pathway and acts as an inflammation inducer.

**Figure 6:**
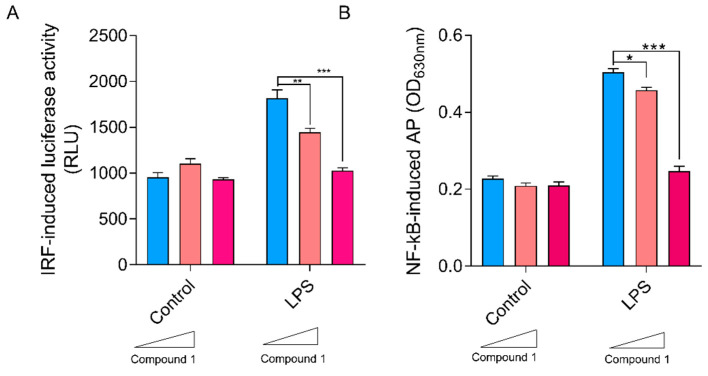
Effect of compound 1 on RAW-Lucia cells. (**A**) Luciferase activity after incubation for 24 h with 0, 1 or 10 µg/mL of compound 1 and LPS (0.5 μg/mL) vs. unstimulated control cells. (**B**) Effect of compound 1 on activation of NF-kB in Raw-Blue cells. Secreted embryonic alkaline phosphatase (SEAP) activity after a 24 h incubation with 0, 1 or 10 µg/mL of compound 1 in treated cells with LPS (0.5 μg/mL) vs. untreated control cells. SEAP activity was determined with the QUANTI-Blue™ reagent Results are expressed as mean ± SEM. * *p* < 0.05, ** *p* ˂ 0.01, *** *p* < 0.001, two-way ANOVA + Tukey post-test.

Results showed that compound 1 did not affect the basal levels of Lucia luciferase activity in control cells, while, when stimulated with LPS, increasing concentrations of the compound were associated with a reduction in enzymatic activity, indicating inhibition of the IFN-I proinflammatory pathway (Figure 6A). 

Considering that activation of TLR4 via LPS interaction culminates in induction of NF-kB and the release of several proinflammatory cytokines, we proceeded to analyze the effect on this proinflammatory transcription factor. For this purpose, the RAW-Blue cell line was used in an in vitro stimulation assay with LPS. This cell line produces secreting embryonic alkaline phosphatase (SEAP) after activation by NF-kB. To determine the effect of compound 1, Raw-Blue cells were cultured with different concentrations of this phytochemical after stimulation with LPS.

As can be seen in Figure 6B, regarding compound 1 upon stimulation with the TLR4 agonist, the presence of increasing concentrations of compound 1 resulted in dose-dependent inhibition of the LPS-mediated activation of NF-kB.

**Figure 7:**
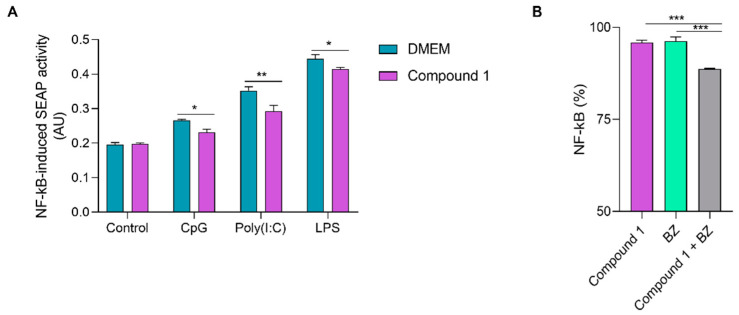
(**A**) Effect of compound 1 on activation of NF-kB in Raw-Blue cells stimulated with TLR (Toll-like receptors) agonists. SEAP activity was determined 24 h after stimulation with CpG (100 μg/mL), Poly(I:C) (100 μg/mL) or LPS (0.5 μg/mL) in the presence or absence of compound 1 (2.5 µg/mL). Blue bars represent SEAP activity of unstimulated control cells. Results are expressed as the means ± SEM. * *p* < 0.05, ** *p* < 0.01, two-way ANOVA + Sidak post-test. (**B**) NF-kB activation on Raw-Blue cells stimulated with LPS after combined treatment with compound 1 and Benznidazole (BZ). Raw-blue cells were treated with compound 1 (2.5 µg/mL), BZ (100µM) or both drugs combined either in the presence or absence of LPS (0.5 μg/mL). After a 24 h incubation, SEAP activity was measured. Results are expressed as NF-kB (%) = (Absorbance _treated LPS_-Absorbance _treated DMEM_)/(Absorbance _control LPS_-Absorbance _control DMEM_) × 100. Results are expressed as means ± SEM. ***, *p* < 0.001. One-way ANOVA+ Tukey post-test.

In order to study whether compound 1 could inhibit the activation of NF-kB induced by other TLR agonists, the secretion of SEAP in cultures of RAW-Blue cells stimulated with either polyinosinic-polycytidylic acid poly(I:C), CpG or LPS as TLR3, TLR9 and TLR4 ligands, respectively, in the presence or absence of the compound, was determined. As depicted in Figure 7A, compound 1 was able to significantly inhibit NF-kB activation in response to stimulation of these TLRs.

Since benznidazole (BZ), the reference drug for the treatment of Chagas disease, has previously been demonstrated to be immunomodulatory over the NF-kB pathway [31], the combination of this drug with compound 1 was studied over the Raw-Blue cell line stimulated with LPS. Results showed a reduction of NF-kB activity in cells stimulated with LPS with the combined treatment of BZ and compound 1 (Figure 7B).

**Figure 8:**
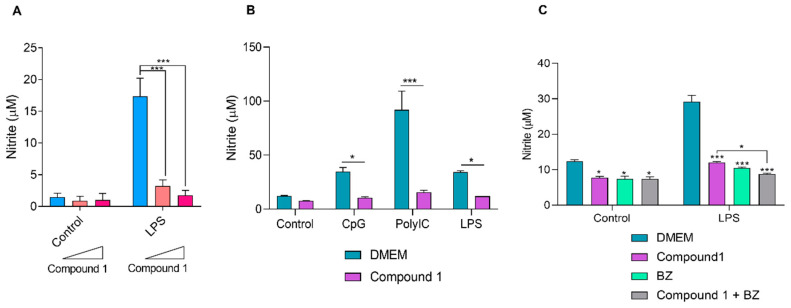
Effect of compound 1 on nitric oxide production. (**A**) Nitrite levels were measured in the supernatants of Raw-Blue cells after stimulation with LPS in the presence of compound 1 using the Griess reagent. Unstimulated cells were used as controls. Results are expressed as means ± SEM. *** *p* < 0.001, two-way ANOVA + Tukey post-test. (**B**) Nitrite levels in culture supernatants 24 h post-stimulation with CpG (100 μg/mL), Poly(I:C) (100 μg/mL) and LPS (0.5 μg/mL) in the presence or absence of 2.5 μg/mL of compound 1. Untreated (control group) and unstimulated cells (DMEM group) were included. Results are expressed as means ± SEM. * *p* < 0.05, *** *p* < 0.001, two-way ANOVA + Sidak post-test. (**C**) Raw-Blue cells treated with 2.5 μg/mL of compound 1, 100 μM of BZ, or their combination, in the presence or absence of LPS (0.5 μg/mL). After 24 h post-stimulation, nitric oxide production was determined. Results are expressed as means ± SEM. * *p* < 0.05, *** *p* < 0.001, two-way ANOVA + Sidak post-test.

Nitrites represent stable end-products of nitric oxide production. Therefore, the Griess reaction was employed to monitor nitrite concentrations in the supernatants of the Raw-Blue cultures, with the different stimuli in the presence or absence of the compound 1. As observed in Figure 8A, the compound significantly inhibited nitric oxide production in a dose-dependent manner when incubated with LPS-stimulated macrophages.

Additionally, we assessed the ability of compound 1 to modulate the production of nitric oxide in the supernatants of Raw-Blue cells stimulated with ligands of TLR3, 9 and 4 (CpG, Poly(I:C) and LPS, respectively). As can be seen in Figure 8B, in the presence of compound 1, cells stimulated with the different TLR ligands exhibited a significant decrease in nitric oxide production, as determined by nitrite quantification.

Subsequently, the production of nitric oxide in culture supernatants of macrophages, treated as previously indicated, upon stimulation with LPS followed by treatment with BZ, compound 1, or their combination was analyzed. A significant decrease in nitrite levels with the combined treatment was observed comparing the levels of nitrites registered with treatment with compound 1 alone (Figure 8C).

**Figure 9:**
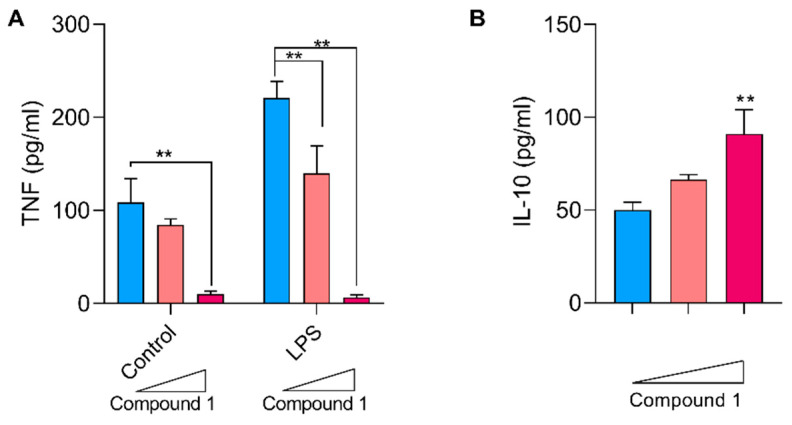
Effect of compound 1 on the production of cytokines in Raw-Blue macrophages. Cells were cultured in the presence of increasing concentrations of compound 1 (0, 1, or 10 μg/mL) with or without LPS stimulation (0.5 μg/mL). After 24 h, the supernatants were collected. (**A**) Levels of TNF in culture supernatants evaluated using capture ELISA. The results are expressed as means ± SEM. ** *p* < 0.01, two-way ANOVA + Tukey post-test. (**B**) Levels of IL-10 in cultures of Raw-Blue cells treated with increasing concentrations of compound 1 determined by an ELISA assay. ** *p* < 0.01, one-way ANOVA + Dunnett post-test.

Given the inhibitory effect on the NF-kB pathway displayed by compound 1, we assessed the secretion of TNF as a model pro-inflammatory cytokine. It was observed that incubation with increasing concentrations of compound 1 led to a significant decrease in the basal levels of TNF upon treatment with compound 1. Similarly to what was observed for NF-kB activation, LPS stimulation resulted in an increase in TNF levels, which was significantly reduced by the addition of 1 and 10 μg/mL of compound 1 (Figure 9A). The production of IL-10 as an immunoregulatory cytokine was also determined. For this purpose, Raw cells were treated with increasing concentrations of compound 1, and IL-10 levels were quantified via capture ELISA. The presence of the compound induced an increase in the production and secretion of IL-10 after a 24 h incubation, which was dose-dependent (Figure 9B). 

### 3.6. In Vivo Assay in T. cruzi Murine Model

Based on the high activity and selectivity demonstrated by compound 1 against *T. cruzi* in vitro, this phytochemical was selected for in vivo evaluation in a murine model of experimental *T. cruzi* infection. Animals were infected with 1000 bloodstream trypomastigotes of *T. cruzi* (RA strain), to be treated with compound 1, BZ, or their combination (1 mg/kg/day intraperitoneally each) for 5 consecutive days. The evolution of parasitemia over time was evaluated. Additionally, the body weight and survival time of the animals were recorded.

**Figure 10:**
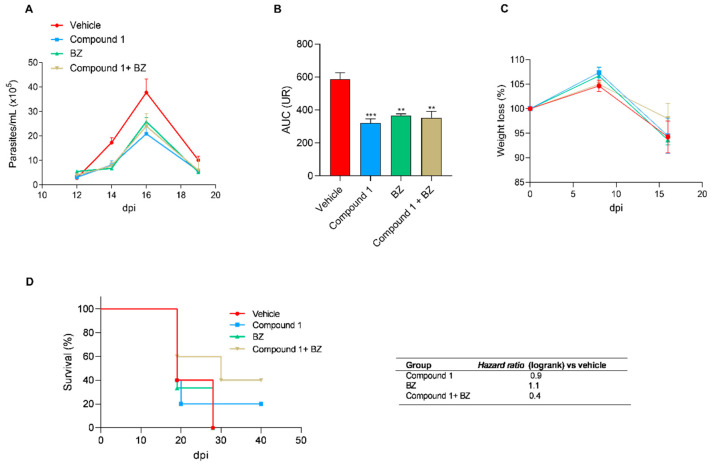
Efficacy of treatment with compound 1 on a murine model of *T. cruzi* infection. Female Balb/c mice were infected and then treated with Vehicle (placebo group), compound 1, Benznidazole (BZ) or a combination of Benznidazole and compound 1 for 5 days with a dose of 1 mg/kg/day, intraperitoneally. (**A**) Parasitemia over time. (**B**) Area under the parasitemia curve (AUC). Parasitemia was measured weekly via direct counting in a Neubauer chamber. Results are expressed as means ± SEM, ** *p* < 0.01, *** *p* < 0.001. (**C**) Weight loss. Results are expressed as a percentage of the initial weight at day 0. (**D**) Kaplan–Meier survival curve for the groups analyzed. The hazard ratio for each group is shown in the table as an indicator of the probability of a near-death event vs. the control group (vehicle).

As observed in Figure 10A, treated animals showed a reduction in parasitemia levels compared to untreated control mice that received only vehicle (0.06 mL/kg DMSO). Regarding compound 1, approximately a two-fold decrease in the area under the parasitemia curve (AUC) was observed in treated animals compared to the control group. For the groups treated with BZ and the combination of BZ and compound 1, the decrease in AUC was 1.6 and 1.7, respectively, compared to the untreated control group (Figure 10B).

No significant changes were observed in the weight loss of the treated animals (Figure 10C). Our findings revealed a significant improvement in survival for mice receiving the combined treatment. At the end of the acute infection phase, 40% of mice in this group remained alive, compared to none in the groups receiving only BZ or the vehicle (Figure 10D). Furthermore, the combined treatment group exhibited the lowest hazard ratio (0.4), indicating a 2.5-fold reduction in the risk of death compared to the control group.

## 4. Discussion

One of the approaches in the drug discovery process for neglected tropical diseases, such as Chagas disease, is the search for novel bioactive molecules from natural sources [32]. In this sense, we have previously reported the anti-*T. cruzi* activity of extracts of several Asteraceae species. Among them, the dichloromethane extract of *Gymnocoronis spilanthoides* var. *subcordata* showed promising activity [19]. In the present study, the fractionation of the active extract and isolation of compounds was carried out. Three diterpenoids from *ent*-kaurane type, namely *ent*-9α,11α-dihydroxy-15-oxo-kaur-16-en-19-oic acid or adenostemmoic acid B (compound 1), (16*R*)-*ent*-11α-hydroxy-15-oxokauran-19-oic acid (compound 2) and *ent*-11α-hydroxy-15-oxo-kaur-16-en-19-oic acid (compound 3), were isolated. Adenostemmoic acid B was previously isolated from *Adenostemma lavenia* (Asteraceae) [21]. The diterpene (16*R*)-*ent*-11α-hydroxy-15-oxokauran-19-oic acid (compound 2), a 16,17-dihydroderivative of compound 3 was previously found in *Eupatorium álbum* [30] and *Adenostemma caffrum* [33], while *ent*-11α-hydroxy-15-oxo-kaur-16-en-19-oic acid, (compound 3) has been reported in *Eupatorium album* [30], *Adenostemma caffrum* [33] and *Adenostemma lavenia* [34].

When anti-*T. cruzi* in vitro activity of the isolated diterpenes was assayed, adenostemmoic acid B was the most active and selective compound. According to bibliographic sources, the exo-methylene cyclopentenone system in the D ring of the diterpenoids would be responsible for the activity observed in these types of structures. The exomethylene group plays a very valuable role due to its ability to alkylate biological nucleophiles. Added to this, divergences in molecular conformation could affect steric accessibility to Michael addition sites, as well as the lipophilicity and polarization of the molecule [35]. Compounds 1 and 3 have the same structure and differ only in the presence or absence of a hydroxyl group at C9. This difference has a significant effect against the intracellular form of the parasite, since compound 1 is ten times more active than compound 3. Comparing compound 2 and 3, the former lacks the double bond C16-C17 which apparently was not crucial for activity against the intracellular form of *T. cruzi*. The presence of a hydroxyl group at C9 would be responsible for the higher activity observed for adenostemmoic acid B (compound 1), and would play a critical role in anti-amastigote activity. These observations are in accordance with what was observed for the antiproliferative activity of this group of compounds [36]. The results obtained in this research are comparable to those reported for other diterpenes of the *ent*-kaurane type, where, in general terms, it is observed that diterpenes are less active against *T. cruzi* trypomastigotes compared to the intracellular form of this protozoan [37,38].

Adenostemmoic acid B was active against the proliferative stages of *T. cruzi,* epimastigotes and amastigotes. This compound was also the least cytotoxic on mammalian cells and presented the highest selectivity index against *T. cruzi*. In view of these results, this compound was selected for further studies. In this sense, the effect of adenostemmoic acid B on the ultrastructure of *T. cruzi* was evaluated. The structural changes observed via TEM after treatment of parasites with adenostemmoic acid B suggest that the anti-*T. cruzi* activity of compound 1 seems to be related to the induction of autophagy, at least in the epimastigote stage of the parasite. As has been reported, the formation of myelin-like structures is a frequent observation in stress parasites upon drug treatment and is associated with the autophagy process [39,40]. 

Chagas disease is a chronic infection that goes unnoticed in most cases. However, approximately 30% of infected patients progress to either the cardiac or digestive form of the disease. This symptomatic stage is associated with an imbalanced inflammatory response with higher levels of TNF and other proinflammatory markers [41]. Current drugs for Chagas disease are effective mainly in the acute stage of the infection, but fail to stop the progress of the disease during the chronic stage. In that sense, studying the mechanisms of drug immunoregulation is of interest to improve host protection by controlling inflammatory reactions. Macrophages represent one of the first cellular types that interact with *T.* c*ruzi* upon infection. As a phagocyte, it has a dual role: it can contribute to parasite elimination and inflammation in the case of M1 polarized cells or promote tissue repair and fibrosis in the case of M2 macrophages [42]. 

The presence of adenostemmoic acid B inhibited IRF-induced luciferase secretion, indicating an inhibitory effect on IFN-I related pathways. The role of IFN-I is still debated in Chagas disease. In experimental murine models, it has been shown that IFN-I increases susceptibility to infection in scenarios where parasite loads are high, since they impact negatively on production of IFN-γ, limiting infection [43]. Recently, an increase in the expression of the antiviral response, which includes the production of IFN-I, in human dendritic cells after contact with metacyclic trypomastigotes of *T. cruzi* has been demonstrated, indicating the importance of this pathway during the first moments of infection [44]. In line with our results, adenostemmoic acid B has recently been shown to inhibit iNOS expression and NO production in vitro [45]. We showed that the anti-inflammatory effect of adenostemmoic acid B was associated with an inhibition of the activity of transcription factors of the NF-kB and IRFs families, indicating a pleiotropic effect of the compound. 

Treatment of LPS-stimulated macrophages with adenostemmoic acid B was able to decrease TNF secretion and nitric oxide production. It has been observed in patients during the chronic phase of Chagas disease that high levels of TNF are associated with poor cardiac function [12]. Furthermore, the implication of TNF and transforming growth factor beta (TGF-β) in Chagas cardiomyopathy has been pointed out. The presence of TNF has been observed in histopathological studies in the hearts of patients who died from Chagas cardiomyopathy. Furthermore, a high number of cells producing IFN-γ or TNF has been demonstrated in autopsy samples, which has been associated with the appearance of cardiac dysfunction [46].

In the chronic phase of Chagas disease, IL-10 participates in delaying the onset of chronic Chagas heart disease (CCD) in infected individuals. A comprehensive analysis of the cytokine profile revealed that the decrease in IL-10 levels shifts the immune response from the anti-inflammatory profile of asymptomatic patients to that of cardiac patients [11]. Considering the anti-inflammatory potential of the compound in macrophage cell lines, we decided to evaluate the secretion capacity of IL-10 as an immunoregulatory cytokine. The compound adenostemmoic acid B induced an increase in the production and secretion of IL-10 after 24 h of culture, which was dose-dependent and was associated with the aforementioned anti-inflammatory effect. In this way, considering that IL-10 plays a key role in modulating the immune response and that Chagas disease is characterized by a sustained inflammatory response that can cause cardiac dysfunction, development of new drugs that can induce the production of this cytokine in infected patients could have benefits such as preventing progression towards symptomatic forms or even contributing to reversing existing pathology [47]. 

Moreover, we also evaluated the effect of adenostemmoic acid B on RAW-Blue cells, stimulated by other TLR agonists. The compound was able to significantly inhibit activation of NF-kB upon stimulation of other TLRs, indicating that its activity is independent of the specificity of the receptor and that it might be targeting a shared protein of these pathways. The TLR9 receptor is one of the members of the TLR family located in the endolysosomal subcellular compartment and can recognize parasite-derived DNA sequences [48]. Stimulation of TLR2 and TLR4 in symptomatic patients, particularly those with the cardiac form, leads to the preferential production of pro-inflammatory cytokines in contrast to anti-inflammatory ones in indeterminate patients [49]. Therefore, reducing the activity of these receptors could have a positive effect on the treatment of Chagas disease.

The combination of adenostemmoic acid B with benznidazole showed greater inhibition of NF-kB and a decrease in nitrite concentration compared to treatment with the compound or benznidazole alone. This information allows us to infer that the combination would have a synergistic effect, pending its in vivo validation.

Adenostemmoic acid B was active in a murine model of *T. cruzi* infection, reducing parasitemia and weight loss of animals during the acute phase of infection. Treatment with this diterpene associated with benznidazole increased the survival time of the animals. Interestingly, lethality in this acute model of Chagas disease is usually associated with an exacerbated inflammatory response related to high levels of parasite burden observed [50,51]. In that sense, the anti-inflammatory activity of the compound might be contributing to this observation.

Future efforts will be focused on the evaluation of adenostemmoic acid B or its derivatives’ abilities to prevent the tissue damage associated with chronic *T. cruzi* infection.

## 5. Conclusions

In this paper we report for the first time the isolation and identification of three diterpenoids of the *ent*-kaurane type from the bioactive extract of *Gymnocoronis spilanthoides* var. *spilanthoides*. Among them, adenostemmoic acid B was the most active and selective compound in vitro on *T. cruzi*. This compound presented an anti-inflammatory effect in vitro in murine macrophages stimulated with LPS and other TLR agonists such as CpG and poly(I:C). The anti-inflammatory effect of adenostemmoic acid B was associated with an inhibition of the activity of transcription factors of the NF-kB and IRFs families. Treatment of macrophages with adenostemmoic acid B was able to reduce TNF secretion, nitric oxide production and increase IL-10 production. The combination of adenostemmoic acid B with benznidazole presented a greater inhibition of NF-kB and a decrease in nitrite concentration in comparison with the compounds used separately. Adenostemmoic acid B was active in a murine model of *T. cruzi* infection, reducing parasitemia and weight loss of animals during the acute phase of the infection. Treatment with this diterpene associated with benznidazole increased the survival time of the animals. These results demonstrate that adenostemmoic acid B is a bioactive diterpene that might be a good lead for the development of new anti-*Trypanosoma cruzi* drugs. 

## Figures and Tables

**Table 1:** NMR data of compound 1 in DMSO-d_6_.

Position	δ ^1^H (*J* Hz) *	δ ^13^C (HSQC)	H-H COSY	NOESY	HMBC	Reported ^Ʊ^ Pyridine-d_5_
1a ^a^ 1b	1.86ddd (12, 12, 4) 1.22 d br (12)	31.6 CH_2_	1b, 2a, 2b 1a, 2a, 2b, 3a (W)	1b 1a, 11	20, 3a	32.7 ^¥^
2a 2b	1.79 m 1.34 m	18.5 CH_2_	1a,1b, 2b, 3a, 3b 1a, 1b, 2a, 3a, 3b	--- ---	1a, 3b	19.8
3a 3b	1.96 d br (13) 0.93 td (13, 4)	37.4 CH_2_	2a, 2b, 3b, 1b (W) 2a, 2b, 3a	3b, 18 3a	18, 1b	38.6
4	---	42.9 C	---	---	18, 3b	44.5
5	1.77 (overlapped m)	48.6 CH	6a,b	---	20, 18, 3a, 7a, 7b	50.0
6a 6b	1.72–1.82 (overlapped m)	20.0 CH_2_	5, 6b, 7a, 7b 5, 6a, 7a, 7b	--- ---	7a, 5	21.2
7a	2.13 m	29.6 CH_2_	6a,b, 7b	---	5, 6a,b	31.0 ^¥^
7b	0.97 m		6a,b, 7a	---		
8	---	55.7 C	---	---	14a, 7a, 7b, 14b, 6a,b	56.9
9	---	77.9 C	---	---	20, 14a, 14b, 12b, 11	79.5
10	---	43.4 C	---	---	20, 6a,b	44.4
11	3.77 dd *br* (3.8, 2)	64.8 CH	12a,C_11_-OH	1b, 12a, 12b	13, 12b	66.3
12a	2.13 m	41.6 CH_2_	11, 13, 12b	---	14a, 14b	42.7
12b	2.06 d br (14)		11, 13, 12a, 14b (W)	12a, 11, 13		
13	2.92 m	35.9 CH	12a, 12b, 14b	14a,12a, 12b	14a, 17a, 17b	37.4
14a	2.53 d (12)	37.3 CH_2_	14b	20, 14b	12a, 7a	38.6 ^¥^
14b	1.32ddd (12, 4, 3)		13, 14a, 12b (W)	14a		
15	---	206.1 C	---	---	14a, 17a, 17b	207.3
16	---	150.4 C	---	---	12a, 14a, 17a, 17b	151.6
17a	5.52 s	110.8 CH_2_	17b	17b	14a	111.1
17b	5.15 s		17a	17a		
18 ^b^	1.12 s	28.8 CH_3_	---	---		29.7
19	---	178.9 C	---	---	3a, 5, 18	180.4
20 ^b^	1.01 s	16.6 CH3	---	2a, 14a	1a, 5	17.6

Others: 5.58 d (3.8; C_11_-OH) and 3.69 s (C_9_-OH). * Most of the signals show broadening owing to the loss of fine coupling caused by the solvent viscosity. ^a^ Assignments based on HSQC, COSY, NOESY and HMBC experiments in DMSO-d6 at 600 MHz for ^1^H and 125 MHz for ^13^C; chemical shifts are relative to TMS; coupling constants are in Hz. ^b^ Intensity of three protons. ^Ʊ^ See [29]. ^¥^ Assignment revised according to this work.

**Table 2:** NMR data of compound 2 and 3 in CDCl_3_.

Position	Compound 2		Compound 3	
	δ _C_	δ _H_ (J in Hz)	δ _C_	δ _H_ (J in Hz)
1	39.7 t		39.5 t	
2	18.8 t		18.7 t	
3	37.7 t		37.7 t	
4	43.6 s		43.6 s	
5	55.9 d	1.25 m *	56.0 d	
6	19.9 t		20.0 t	
7	36.6 t		37.2 t	
8	50.5 s		51.0 s	
9	63.1 d	1.40 s *br*	62.9 d	
10	39.0 s		38.7 s	
11	66.3 d	4.05 d *br* (4.8)	65.3 d	3.93 d *br* (5.6)
12a	41.2 t	2.11 ddd (14.5, 4.8, 3.1)	33.2 t	
12b		1.98 m *		
13	36.9 d	3.06 m	34.8 d	2.44 m
14a	33.7 t	2.37 d (12)	34.5 t	2.35 d (12)
14b		1.42 *		
15	209.6 s		222.5 s	
16	150.2 s		49.5 d	2.28 quint (6.8)
17a	113.0 t	5.87 s	11.1 q	1.25 ^§^ d (6.8)
17b		5.27 s		
18 ^§^	28.9 q	1.28 s	28.8 q	1.26 s
19	181.1 s		176.3 s	
20 ^§^	15.6 q	0.95 s	15.3 q	0.92 s

* Overlapped signal; ^§^ Intensity of three protons.

**Table 3:** IC_50_ values calculated for each compound against the epimastigotes and amastigote forms of *T. cruzi*.

Compounds	IC_50_ ± SEM µg/mL (µM)	
	Epimastigotes	Amastigotes
1	3.7 ± 0.5 (10.6)	2.1 ± 0.3 (6.1)
2	5.2 ± 0.3 (15.9)	6.5 ± 0.2 (19.5)
3	1.6 ± 0.6 (4.8)	20.1 ± 8.2 (60.6)
Benznidazole	1.08 ± 0.2 (4.2)	0.5 ± 0.04 (1.9)

**Table 4:** CC_50_ values and selectivity indexes calculated for each compound for epimastigotes and amastigotes *T. cruzi*.

Compound	CC_50_		SI	
	µg/mL ± SEM	µM	Epimastigotes	Amastigotes
1	112.0 ± 3.4	321.8	30.3	52.7
2	7.8 ± 0.2	23.3	1.5	1.2
3	4.9 ± 1.6	14.8	3.1	0.2
Benznidazole	45.3 ± 1.8	81.5	19.6	42.4

## Data Availability

Data are contained within the article and Supplementary Materials.

## Supplementary Materials

The following supporting information can be downloaded at https://www.mdpi.com/article/10.3390/pharmaceutics16030415/s1: Figure S1: ^1^H-NMR spectrum of compound 1; Figure S2: ^13^C-NMR spectrum of compound 1; Figure S3: HSQC spectrum of compound 1; Figure S4–S6: HMBC spectrum of compound 1; Figure S7: ^1^H-^1^H COSY spectrum of compound 1; Figure S8: NOESY spectrum of compound 1; Figure S9: ^1^H-NMR spectrum of compound 2; Figure S10: ^13^C-NMR spectrum of compound 2; Figure S11: ^1^H-NMR spectrum of compound 3; Figure S12: ^13^C-NMR spectrum of compound 3; Figure S13: Cytotoxicity of benznidazole on murine splenocytes.
