# Supplementary material for: Trypanocidal and Anti-Inflammatory Effects of Three ent-Kaurane Diterpenoids from Gymnocoronis spilanthoides var. subcordata (Asteraceae)
Source: Pharmaceutics. 2024 Mar 18;16(3):415. doi: 10.3390/pharmaceutics16030415 (PMC10975076; doi:10.3390/pharmaceutics16030415)
Supplement: Supplementary file 1 [file pharmaceutics-16-00415-s001.zip › pharmaceutics-2827389-supplementary.pdf]

Trypanocidal and immunomodulatory effects of diterpenoids from *Gymnocoronis spilanthoides* var. *subcordata* (Asteraceae)

Mariana G. Selener [1,2,ψ], Jimena Borgo [1,2,ψ], Maria Belen Sarratea[3,ψ], Maria Alicia Delfino[4] , Laura C. Laurella[1,2], Natacha Cerny[5], , Jessica Gomez[6], Mauro Coll[6], Emilio L. Malchiodi[3,4], Augusto E. Bivona[3,4], Patricia Barrera[6], Flavia C. Redko[1,2], César A. N. Catalán[7], Andrés Sánchez Alberti.[3,5,†,*] and Valeria P. Sulsen [1,2,†,*]

[1] Universidad de Buenos Aires, Facultad de Farmacia y Bioquímica, Cátedra de Farmacognosia, Junín 956, piso 2, Buenos Aires C1113AAD, Argentina.
[2] CONICET-Universidad de Buenos Aires, Instituto de Química y Metabolismo del Fármaco (IQUIMEFA), Junín 956, piso 2, Buenos Aires C1113AAD, Argentina.
[3] CONICET-Universidad de Buenos Aires, Instituto de Estudios de la Inmunidad Humoral (IDEHU), Junín 956, piso 4, Buenos Aires C1113AAD, Argentina.
[4] Universidad de Buenos Aires, Facultad de Farmacia y Bioquímica, Cátedra de Inmunología, Junín 956, piso 4, Buenos Aires C1113AAD, Argentina Buenos Aires, Argentina.
[5] Universidad de Buenos Aires, Facultad de Medicina, Departamento de Microbiología, Parasitología e Inmunología-IMPAM (UBA-CONICET), Paraguay 2155, C1121ABG Buenos Aires, Argentina.
[6] Facultad de Ciencias Médicas, Instituto de Histología y Embriología "Dr. Mario H. Burgos" (IHEM), Universidad Nacional de Cuyo-CONICET, CC 56, Mendoza 5500, Argentina.
[7] Universidad Nacional de Tucumán, Facultad de Bioquímica, Química y Farmacia, Instituto de Química Orgánica, Ayacucho 471, (T4000INI), San Miguel de Tucumán, Tucumán, Argentina.

ψ These authors contributed equally to this work.
† The work was co-directed by both authors.
* Correspondence: vsulsen@ffyb.uba.ar ; Tel.: +54 (011) 5287-4272 and aalberti@ffyb.uba.ar Tel.: +54 (011) 528-53525.

Supplementary material

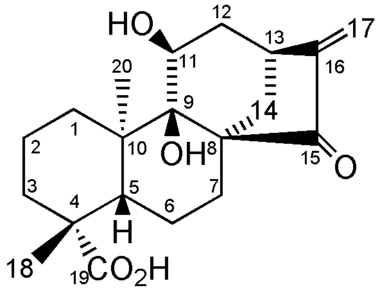

Compound 1

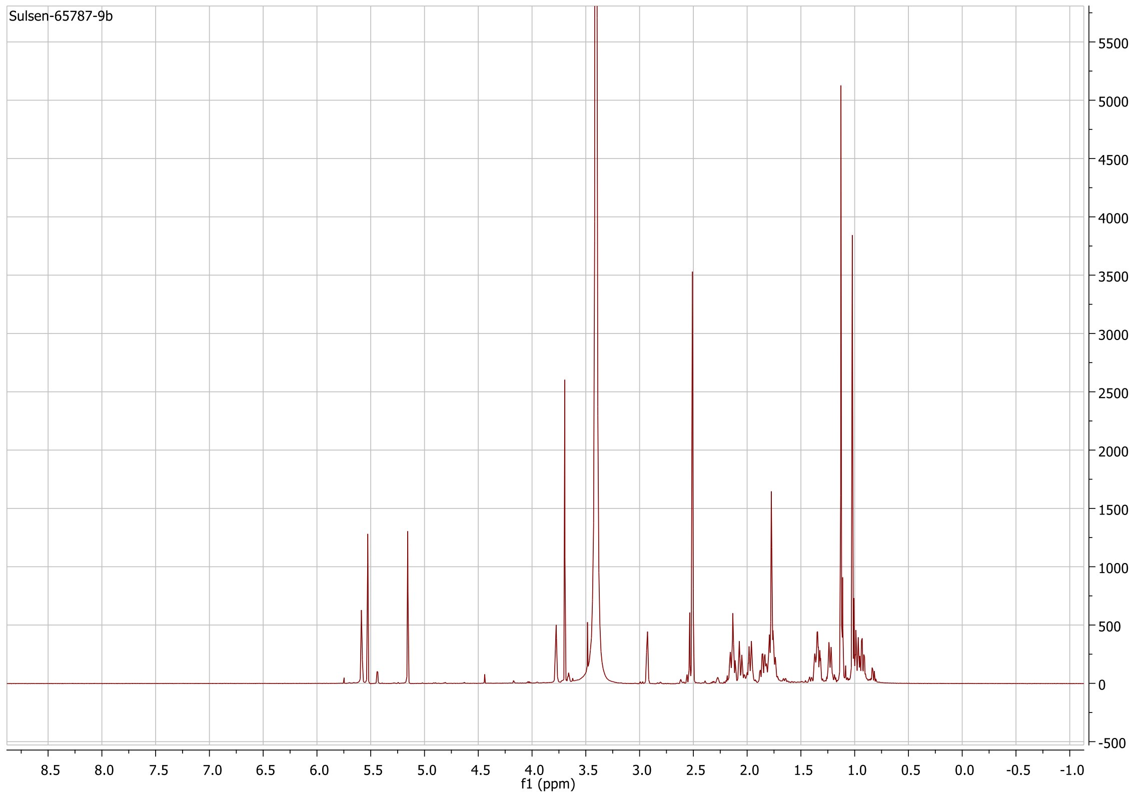

Figure S1. ^{1}H-NMR spectrum of compound **1**

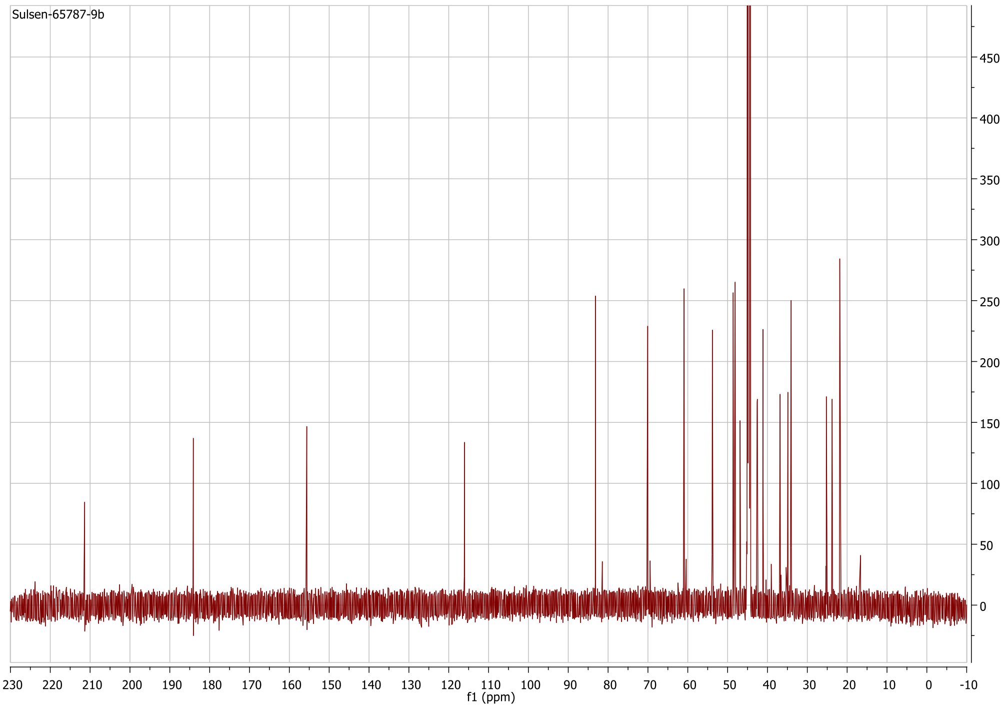

Figure S2. ^{13}C-NMR spectrum of compound **1**

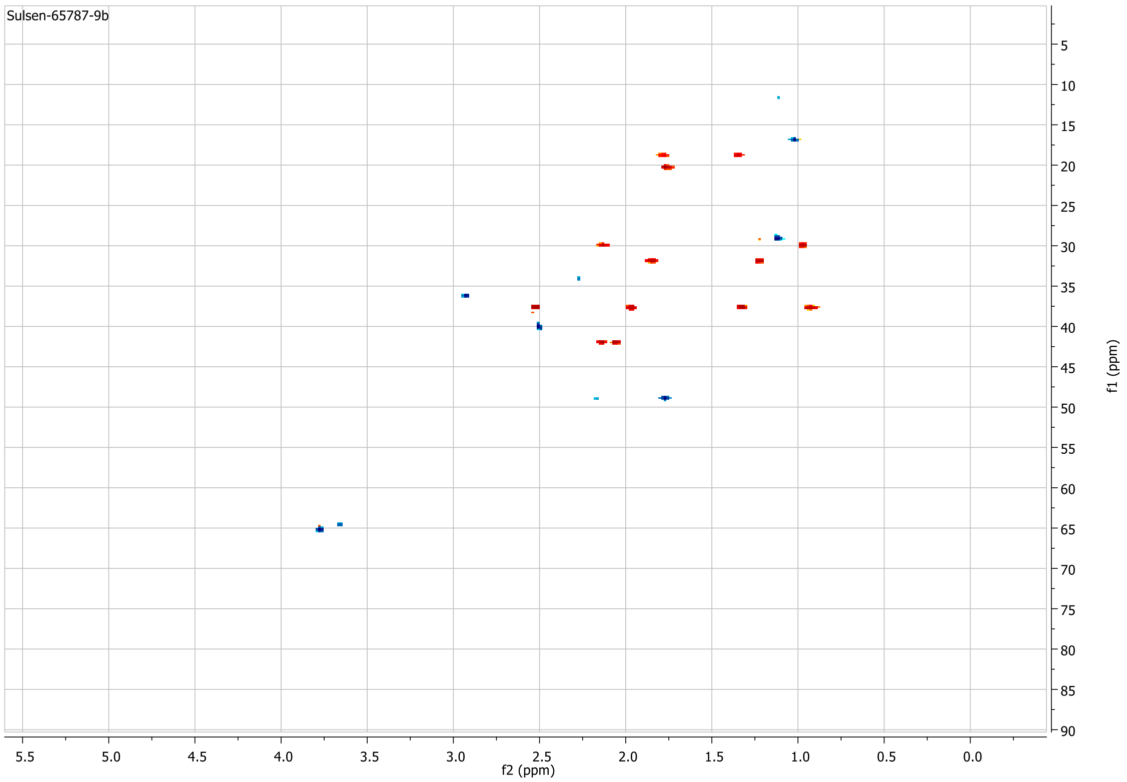

Figure S3. HSQC spectrum of compound **1**

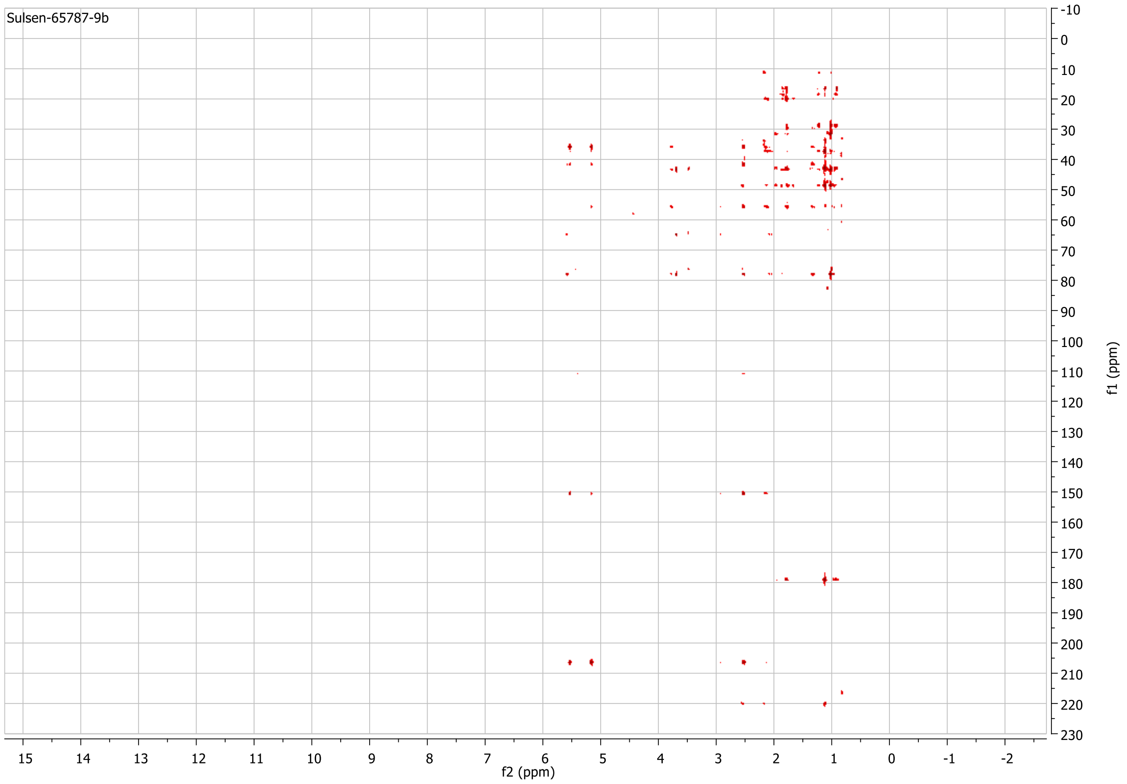

Figure S4. HMBC spectrum of compound **1**

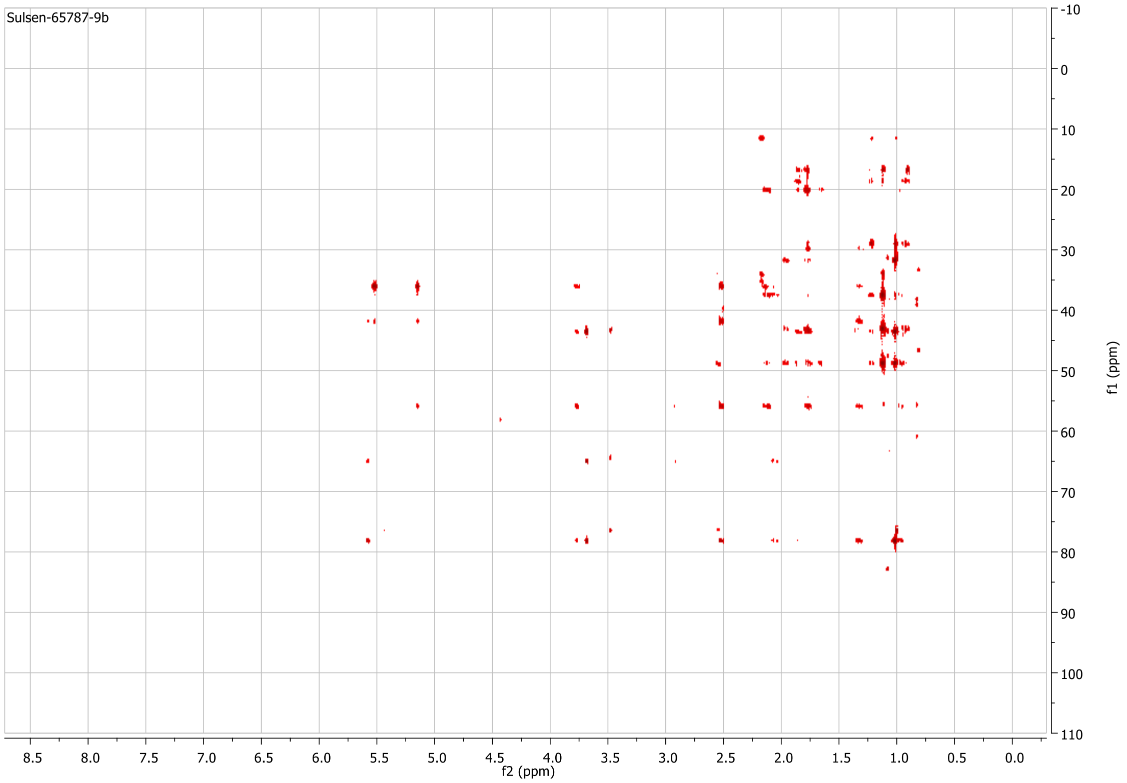

Figure S5. HMBC spectrum of compound **1**

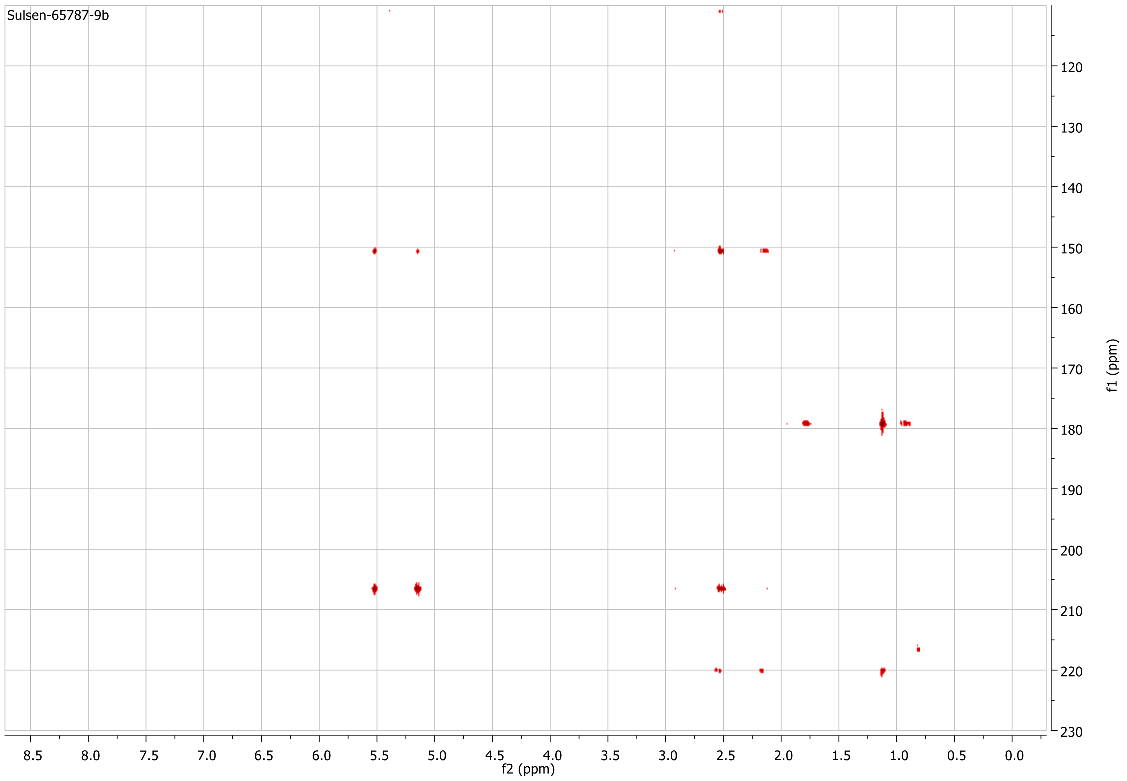

Figure S6. HMBC spectrum of compound **1**

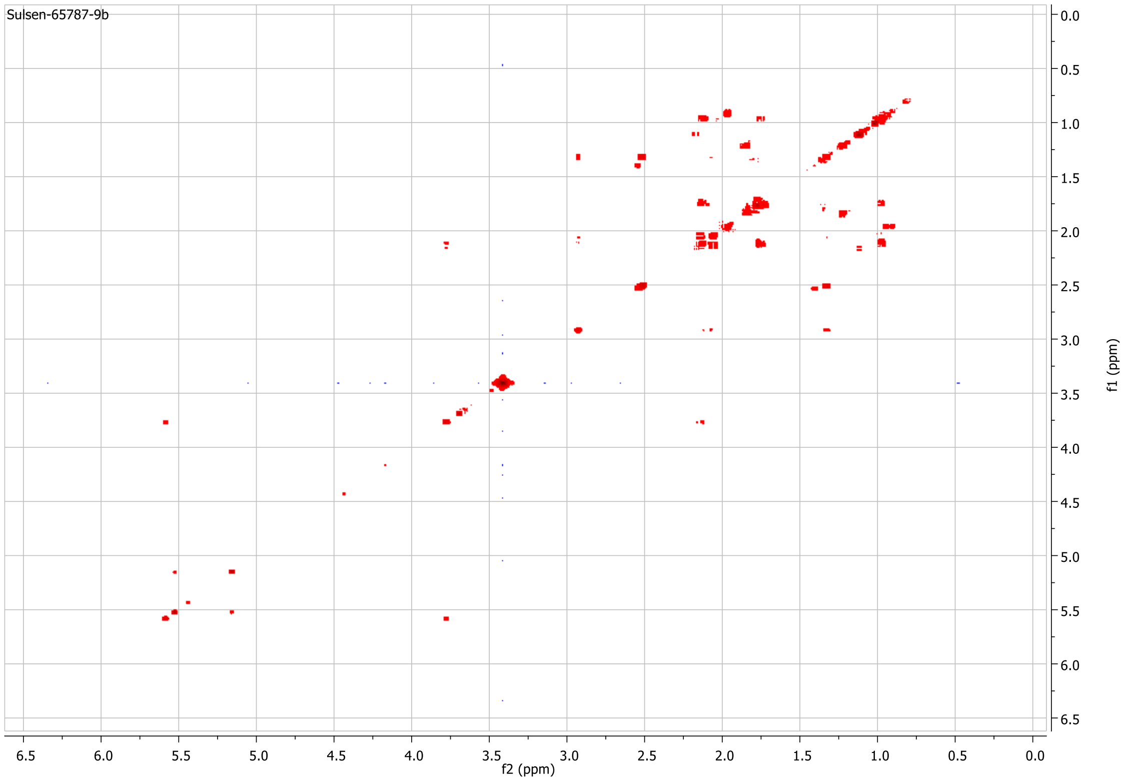

Figure S7. ^{1}H-^{1}H COSY spectrum of compound **1**

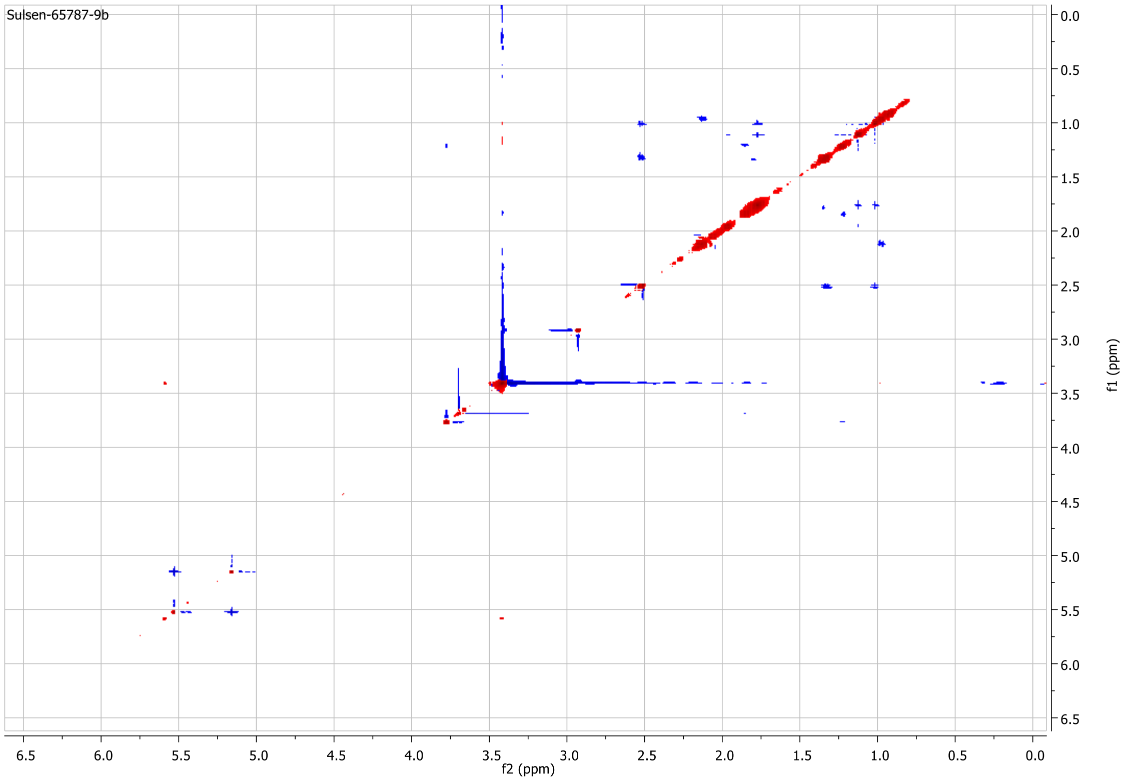

Figure S8. NOESY spectrum of compound **1**

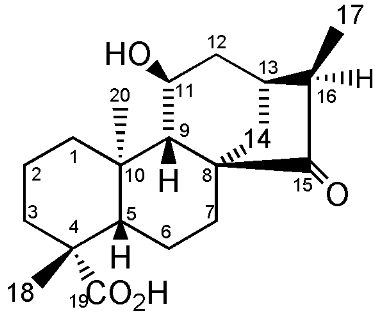

Compound 2

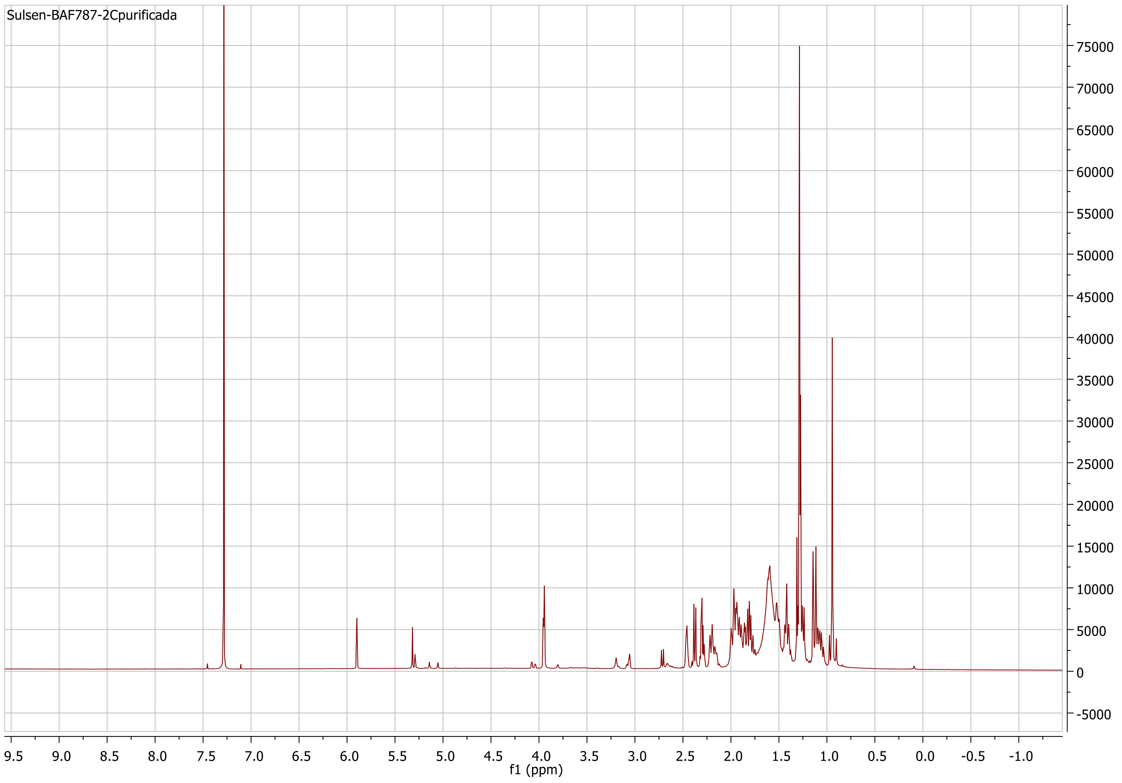

Figure S9. ^{1}H-NMR spectrum of compound **2**

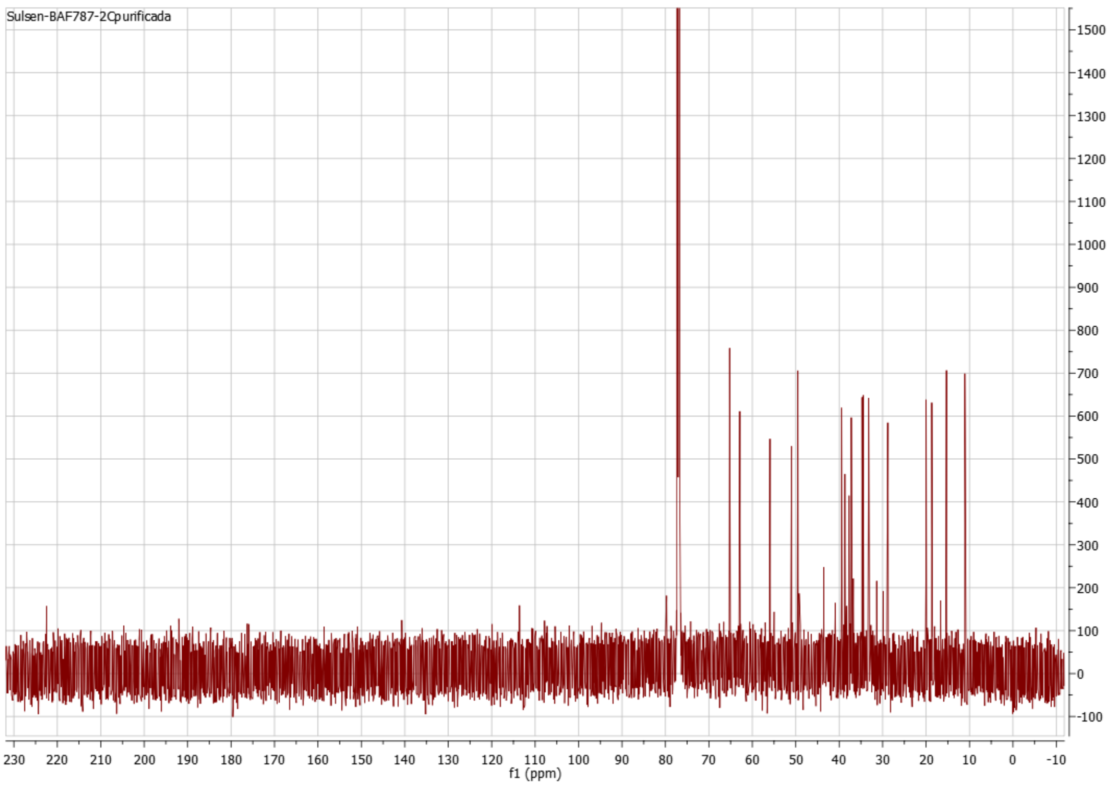

Figure S10. ^{13}C-NMR spectrum of compound **2**

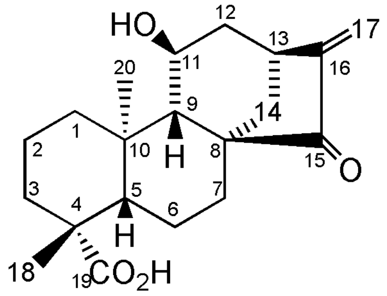

Compound 3

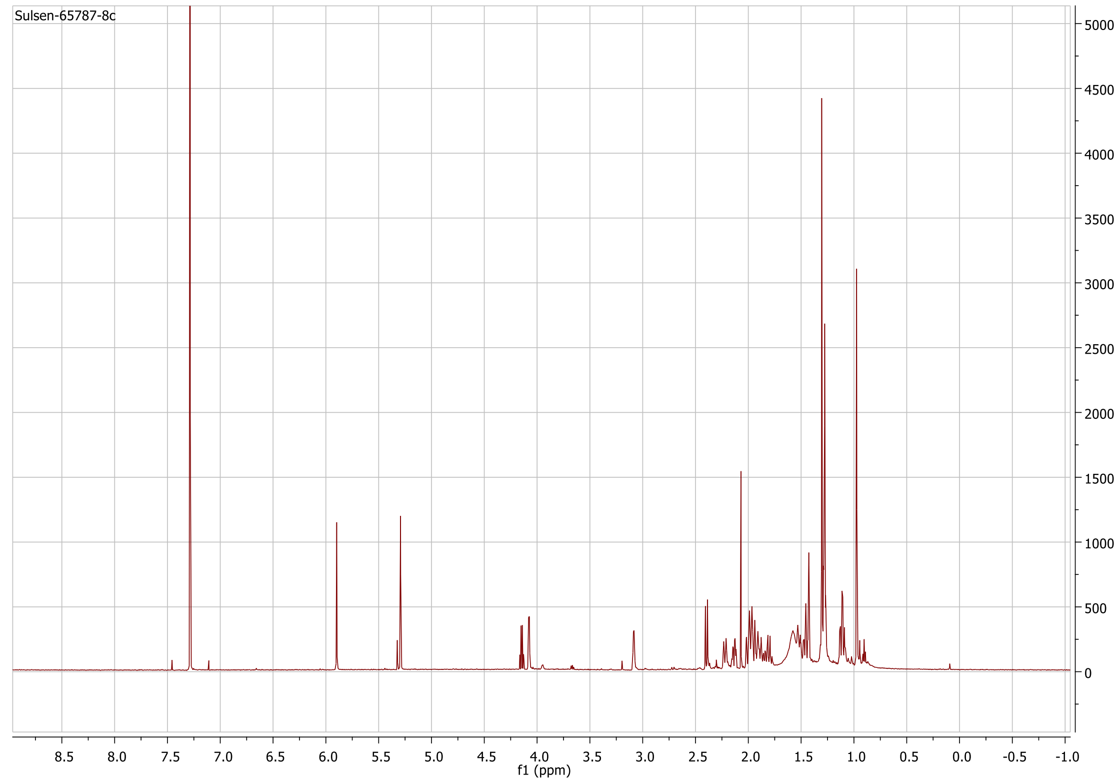

Figure S11. ^{1}H-NMR spectrum of compound **3**

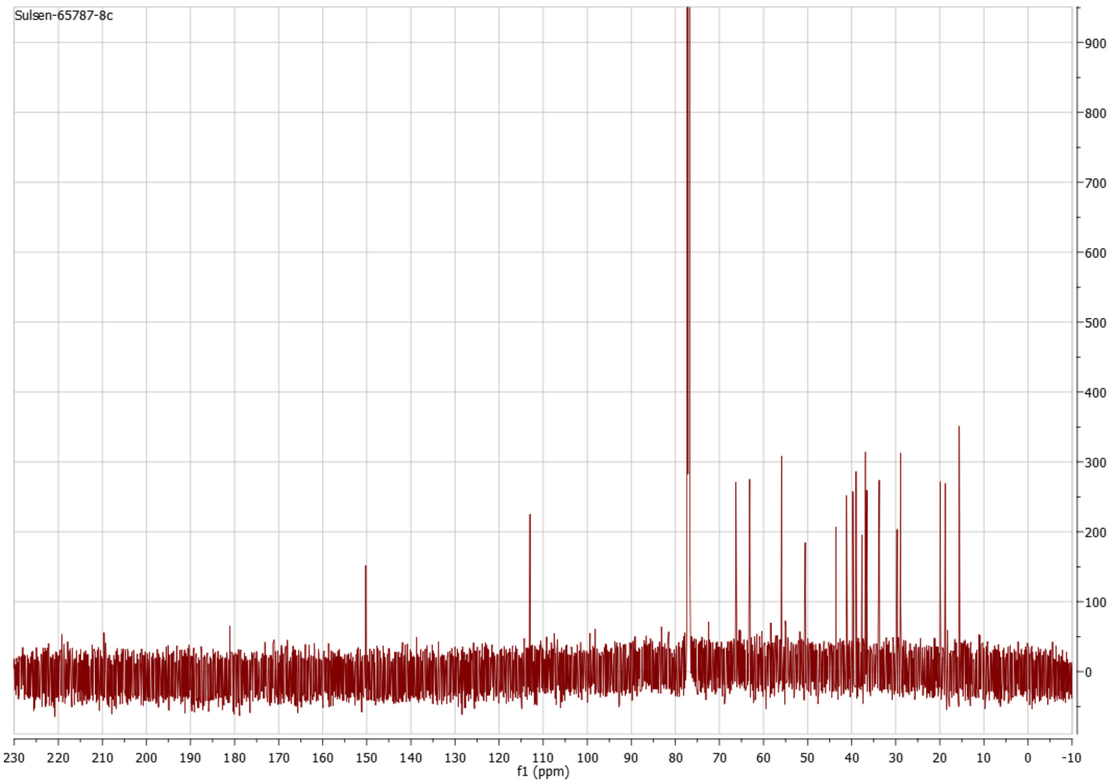

Figure S12. ^{13}C-NMR spectrum of compound **3**

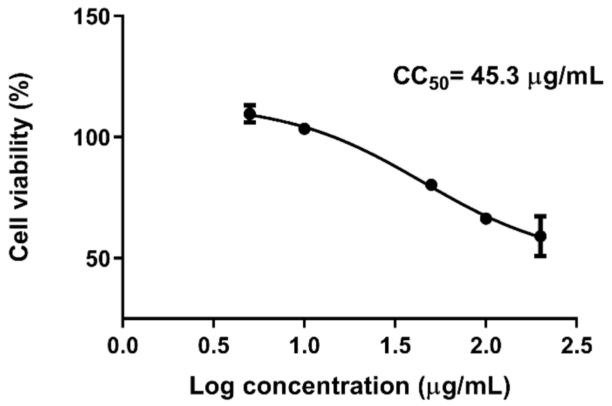

Figure S13. Cytotoxicity of benznidazole on murine splenocytes.
